# *De novo* identification of differentially methylated regions in the human genome

**DOI:** 10.1186/1756-8935-8-6

**Published:** 2015-01-27

**Authors:** Timothy J Peters, Michael J Buckley, Aaron L Statham, Ruth Pidsley, Katherine Samaras, Reginald V Lord, Susan J Clark, Peter L Molloy

**Affiliations:** CSIRO Digital Productivity Flagship, Riverside Life Sciences Centre, 11 Julius Avenue, North Ryde, New South Wales, 2113 Australia; Epigenetics Program, Garvan Institute of Medical Research, Sydney, Australia; St Vincent’s Hospital, Darlinghurst, New South Wales 2010 Australia; School of Medicine, University of Notre Dame, Darlinghurst, New South Wales 2010 Australia; St Vincent’s Clinical School, Faculty of Medicine, University of New South Wales, Darlinghurst, New South Wales 2010 Australia; CSIRO Food and Nutrition Flagship, Riverside Life Sciences Centre, 11 Julius Avenue, Sydney, Australia

**Keywords:** Differential DNA methylation, Kernel smoothing, Illumina

## Abstract

**Background:**

The identification and characterisation of differentially methylated regions (DMRs) between phenotypes in the human genome is of prime interest in epigenetics. We present a novel method, *DMRcate*, that fits replicated methylation measurements from the Illumina HM450K BeadChip (or 450K array) spatially across the genome using a Gaussian kernel. *DMRcate* identifies and ranks the most differentially methylated regions across the genome based on tunable kernel smoothing of the differential methylation (DM) signal. The method is agnostic to both genomic annotation and local change in the direction of the DM signal, removes the bias incurred from irregularly spaced methylation sites, and assigns significance to each DMR called via comparison to a null model.

**Results:**

We show that, for both simulated and real data, the predictive performance of *DMRcate* is superior to those of *Bumphunter* and *Probe Lasso*, and commensurate with that of *comb-p*. For the real data, we validate all array-derived DMRs from the candidate methods on a suite of DMRs derived from whole-genome bisulfite sequencing called from the same DNA samples, using two separate phenotype comparisons.

**Conclusions:**

The agglomeration of genomically localised individual methylation sites into discrete DMRs is currently best served by a combination of DM-signal smoothing and subsequent threshold specification. The findings also suggest the design of the 450K array shows preference for CpG sites that are more likely to be differentially methylated, but its overall coverage does not adequately reflect the depth and complexity of methylation signatures afforded by sequencing.

For the convenience of the research community we have created a user-friendly R software package called *DMRcate*, downloadable from Bioconductor and compatible with existing preprocessing packages, which allows others to apply the same DMR-finding method on 450K array data.

**Electronic supplementary material:**

The online version of this article (doi:10.1186/1756-8935-8-6) contains supplementary material, which is available to authorized users.

## Background

DNA methylation is widely regarded as the most stable epigenetic mark and, for explaining patterns of gene expression, cell differentiation and phenotype, one of the most informative [1–3]. Much interest has focused recently on the development of principled methods for combining information from multiple nearby methylation sites to aid biological inference [4, 5]. Effort has been primarily focused on detecting differentially methylated regions (DMRs). These are contiguous genomic regions that differ between phenotypes [6–8]. DMRs may occur throughout the genome, but have been identified particularly around the promoter regions of genes, within the body of genes, and at intergenic regulatory regions [9–13]. We focus here on DMRs, but it is also of much interest to detect other types of regions, for example, variably methylated regions (VMRs) and regions of hyper- or hypomethylation for unlabelled samples.

The methylation status of a tissue sample can be interrogated at the individual CpG level in two chief ways [14]. Firstly, it can be assayed via whole-genome bisulfite sequencing (WGBS) [15]. This method uses bisulfite conversion and DNA sequencing to assess the methylation status of every CpG dinucleotide in the genome. Secondly, various methods are available for determining the methylation status of specific selected fractions of the genome. One such method is reduced representation bisulfite sequencing [16, 17] in which DNA restriction enzyme digestion and fragment size selection are followed by bisulfite conversion and sequencing. Other methods, such as the Agilent SureSelect^XT^ Human Methyl-Seq and Nimblegen SeqCap Epi System, can be used to target specific sections of the genome for methylation profiling.

Another cost-effective approach is via a microarray specifically designed for a particular genome. For the human genome, one such platform, which is currently very widely used, is the Illumina Infinium Human Methylation 450 BeadChip (hereinafter referred to as the 450K array or simply 450K). This platform uses hybridisation of bisulfite-treated DNA to arrayed probes, combined with a single nucleotide extension to measure methylation at the genomic hybridisation site for a single CpG dinucleotide. Methylation values from the 450K array are shown to have excellent concordance with those from bisulfite sequencing [18–20]. This article will concentrate on data from the 450K array, although the methodology is equally applicable to any genomic assay, reduced representation or WGBS.

The 450K array measures the methylation status of 485,512 methylcytosine sites in the human genome at a single nucleotide resolution, representing approximately 1.5% of total genomic CpG sites [21, 22]. While the assayed CpG sites are concentrated around promoter regions and gene bodies, approximately 25% are located in intergenic regions [21]. The array uses two types of probes (types I and II), each with a different biochemistry. This complicates the preprocessing of the array data, but various normalisation methods are available [23, 24] to remedy this. We assume for this study that any required normalisation and other preprocessing have been carried out before further data analysis.

Usually, knowledge about the methylation status of an individual CpG site is of limited value unless it is contextualised by the status of neighbouring CpG sites. Clusters of hypermethylated CpG sites in the promoter region of a gene are usually associated with silencing of the gene [10], and coordinated hypermethylation in intragenic regions with upregulation [11]. A method informed by spatial information is needed to define and characterise these regions. Many DMR-finding methods are available to the bioinformatics community [25–41]. A good overview of the available methods can be found in Robinson *et al.*[42]. Most are specific to a platform (with the notable exception of *Bumphunter*[25]), and, in addition, many different approaches to incorporating information from neighbouring CpG sites, and controlling the region-wise false discovery rate (FDR), are available. A possible biasing factor is that some genomic regions are more richly annotated than others, which may persuade researchers to concentrate on these at the expense of more enigmatic regions. Methods such as *IMA*[28] and *COHCAP*[29] use pre-annotated regions *a priori*, which comprise only a subset of the 450K probes as the primary backbone for DMR detection, biasing their results. Similarly, QDMR [30] requires genomic regions to be defined by the user prior to evaluation, forcing artificial DMR endpoints. For these reasons, we do not consider this family of methods for our comparison in the Results section. As mentioned, approximately one-quarter of the CpG sites assayed by the 450K array are intergenic, so are simply not accompanied by a gene association in the annotation provided by Illumina. Regions associated with these CpG sites may contain trans-acting enhancers or other regulatory regions [13], and deserve to be considered alongside those with an explicit gene association.

As an alternative, we propose a data-driven approach that is agnostic to all annotations except for spatial ones, specifically chromosomal coordinates. Critical to our method are robust estimates of differential methylation (DM) at individual CpG sites derived from *limma*[43], arguably the most widely used tool for microarray analysis. We pass the square of the moderated *t* statistic calculated on each 450K probe to our DMR-finding function. We then apply a Gaussian kernel to smooth this metric within a given window, and also derive an expected value of the smoothed estimate (in other words, one with no experimental effect) from the varying density of CpGs sites incurred by reduced representation and irregular spacing. *DMRcate* validations were performed on both simulated and real 450K data.

### Simulations

We generated 100 simulated data sets, each with 20 columns and one row for each 450K probe. The first ten columns were considered as control samples and the last ten columns as treatment samples. In each simulated data set, 2,162 promoter-associated regions were randomly assigned as true DMRs, half being hypermethylated in the treatment samples and half hypomethylated. For each of these DMRs, two beta levels were randomly chosen with a beta difference of exactly 0.2. Simulated beta values within the DMR were generated by sampling from a beta distribution with mode equal to the given level, and with a realistic amount of variability. Outside the true DMRs, each probe was classified as unmethylated (beta values near zero) or fully methylated (beta values near 1), based on a representative array produced from healthy human leukocytes. Beta values for these probes were generated by sampling from one of two manually chosen beta distributions, each empirically matching the two main modes in typical 450K arrays derived from leukocyte samples. Finally, values were adjusted to lie in the range 0.01 to 0.99. The logit-transform of the beta value (*M*) was used for all 450K statistical analyses. A more thorough explanation of the data synthesis can be found in Additional file 1: Supplementary material.

### In-house 450K data

With regards to real data, we used two experimental comparisons to validate our method, both designed with paired biological replicates. Methylation of purified visceral adipocytes (VAs) was compared to, firstly, purified subcutaneous adipocytes (SAs) and secondly, to the unpurified tissue from which they were derived, visceral adipose tissue (VAT), matched from the same patients. All DNA samples were taken from three lean, healthy females between the ages of 36 and 47, and form part of the EpiSCOPE research program [44].

**Figure 1:**
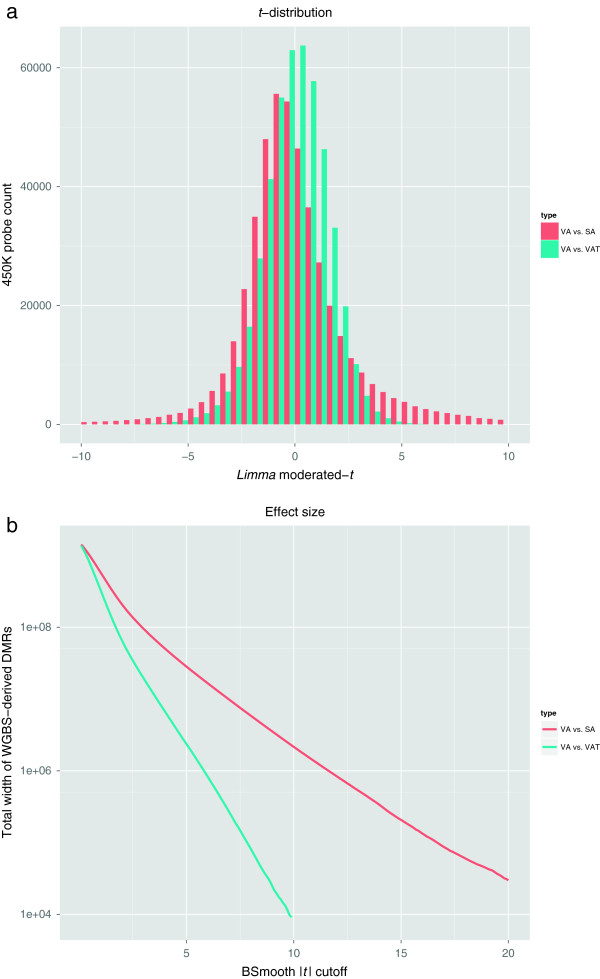
**Effect size for VA vs SA, VA vs VAT comparisons.** **(a)** Moderated *t* distribution of all 450K probes from the VA vs SA and VA vs VAT comparisons, derived from their respective *limma* top tables. **(b)** Log-scaled total width of DMRs called by *BSmooth* on the VA vs SA and VA vs VAT comparisons from WGBS data, for varying values of the |*t*| cutoff. DMR, differentially methylated region; SA, subcutaneous adipocyte; VA, visceral adipocyte; VAT, visceral adipose tissue; WGBS, whole-genome bisulfite sequencing.

For all 450K data, the nine tissue DNA samples were run on the same physical chip, to eliminate any potential for a batch effect between samples. For each comparison, samples were normalised separately using the *dasen* method from the R package *wateRmelon*[23]. Again, *M* values were used for all 450K statistical analyses. To avoid corruption of the analysis from bad quality or confounded data, any row (probe) in the data matrix that contained a detection *P* value (quality control indicator) above 0.05 was discarded, as was any probe whose represented CpG site was two or fewer nucleotides from a known SNP (see Additional file 1: Figure S1) for which that SNP had a minor allele frequency above 0.05. Since all samples were female, cross-hybridising probes (which are predominantly promiscuous on sex chromosomes [45]) and the sex chromosome probes themselves were retained in the analysis. This process was carried out separately for each group of six samples pertinent to the comparison of interest, and resulted in 466,190 candidate methylcytosine sites for the VA vs SA comparison, and 466,263 for the VA vs VAT comparison. The VA vs SA comparison showed a much stronger biological effect than VA vs VAT (Figure 1a) when analysed with *limma*, confirmed by the fact that 71,535 probes (approximately 15% of those assayed) returned a Benjamini–Hochberg (BH) adjusted *P*<0.05 for the former, but none was returned for the latter.

### Whole-genome bisulfite sequence data

**Figure 2:**
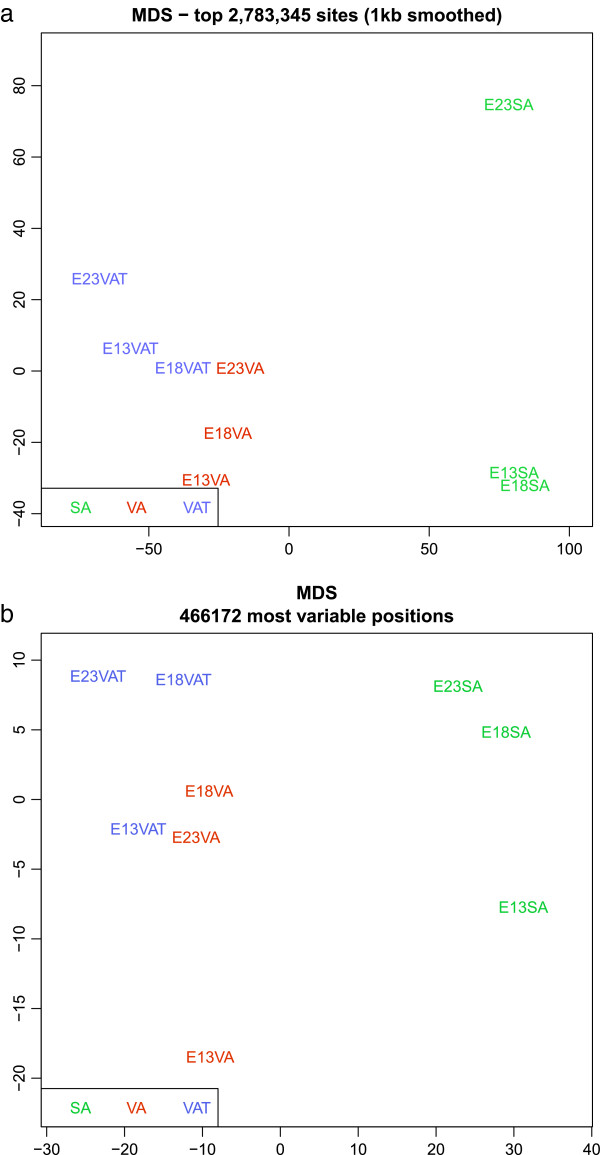
**Multidimensional scaling plots of adipocyte and adipose methylome samples.** **(a)** MDS plot of smoothed WGBS count ratios derived from the nine samples used in this study. **(b)** MDS plot of the matched 450K data from the same DNA samples. Probe count represents the number of probes whose detection *P*<0.05 for all nine samples in the plot. MDS, multidimensional scaling; SA, subcutaneous adipocyte; VA, visceral adipocyte; VAT, visceral adipose tissue; WGBS, whole-genome bisulfite sequencing.

Our 450K samples showed high concordance with WGBS assays on the same DNA, highly preserving their relative position to each other within the first multidimentional scaling (MDS) dimension (Figure 2a,b). Each individual sample, where 450K beta values were compared to sequencing count ratios for matched CpG sites, gave correlation coefficients within the range of 0.95 to 0.96. We obtained good coverage for the majority of CpG sites for all nine samples, with 95% of all genomic CpGs sequenced at a minimum depth of 4, and 90% at a minimum depth of 9 (Additional file 1: Figure S2).

Adipocyte methylomes are largely unexplored, and so in the absence of a ground truth for validation, groupwise DMRs were called from the WGBS assays using the *BSmooth* algorithm [46] and defined as ground truth. More specifically for the sequencing-derived DMRs, a series of values for the argument cutoff (the minimum absolute *t* statistic) was passed to the function dmrFinder() from the Bioconductor package *bsseq*, after CpG sites were filtered for a minimum coverage of two reads. Subsequently, a series of lists of sequencing-derived DMRs for each cutoff was produced, with the values of cutoff passed in increments of 0.1 from 0.1 to 20 for the VA vs SA comparison, and from 0.1 to 10 for VA vs VAT; the difference in maximum thresholds owing to the total DMR coverage in VA vs SA being much larger than that for VA vs VAT (Figure 1b).

### Publicly available data

Finally, for biological provenance of *DMRcate* outputs, we used publicly available 450K assays forming part of a study comparing methylomes of various tissue types from 11 healthy individuals [47] (Gene Expression Omnibus accession [GEO:GSE48472]).

## Results

### Differential methylation by annotation

As mentioned earlier, *DMRcate* intentionally avoids distinguishing between probes that have an explicit gene and/or CpG-island annotation (as provided by Illumina) and those that do not. As evidence of non-trivial DM in genomic regions lacking this annotation, for the 71,535 differentially methylated probes in the VA vs SA comparison, non-CpG-island probes (often referred to as ‘open sea’ probes) were enriched in this subset with an odds ratio of 1.97 compared to their representation on the 450K array, and non-annotated intergenic probes enriched with an odds ratio of 1.59. Despite that these probes are arranged in sparser genomic neighbourhoods on the 450K array, we still see them modestly enriched as constituents of *DMRcate*-derived DMRs for part of the significance threshold domain (Additional file 1: Figure S3a,c). This effect is stronger when the 450K probes are mapped to *BSmooth*-derived DMRs from the WGBS data (Additional file 1: Figure S3b,d).

### Biological inference

To test the outputs of *DMRcate* for biological relevance, we called tissue-specific DMRs for six different tissue types (blood, buccal, liver, muscle, pancreas and spleen) from 450K assays obtained from Slieker *et al.*[47] (see Results) by comparing each individual tissue group to the remaining five tissue groups, accounting for matched samples from the same patients. Gene associations (if any) from the top 150 DMRs for each comparison (all being statistically significant) were tested for functionally enriched gene ontology terms for biological process (GO:BP) using the *goseq* package from Bioconductor [48]. We followed the *goseq* protocol suggested by Geeleher *et al.*[49] to offset the bias incurred by the variable probe count for associations with each represented gene.

Unique ontological terms that were both statistically significant (FDR <0.05) and biologically consonant with the specific tissue tested were found for blood, liver and muscle. For example, significant terms for blood included leukocyte activation (FDR =1.8×10^−7^), immune system process (1.52×10^−5^), lymphocyte activation (1.38×10^−4^), T cell activation (2.54×10^−3^) and B cell activation (4.12×10^−2^). Liver-associated terms included complement activation (2.6×10^−4^), lipid homeostasis (5.86×10^−3^) and cholesterol efflux (7.09×10^−3^). Five of the six muscle-associated significant terms were unequivocally muscle specific, including muscle structure development (1.26×10^−2^) and myofibril assembly (4.76×10^−2^). No significant terms were found for buccal cells, but highly ranked biologically relevant terms such as morphogenesis of an epithelium (ranked fourth out of 12,447 total terms) and epithelium development (ranked 6th) were obtained. A similar profile was observed for pancreas-associated terms, including exocrine pancreas development (ranked seventh) and type B pancreatic cell fate commitment (ranked 16th). Ranked lists of GO:BP terms for each tissue can be found in Additional file 2: Table S1.

### Competing methods

To assess the performance of *DMRcate* relative to three other DMR-finding methods, we looked for other methods whose implementations either satisfied, or were versatile enough to make use of, the following criteria:

- Usage of *limma*-derived statistics for calculation of individual CpG site methylation differences.
- Ability to assess *all* 450K probes as candidates for DMR constituents.

The three other candidate DMR-finding methods we tested were:

- *Bumphunter*[25]: A method that also uses smoothed methylation values to detect DMRs
- *comb-p*[26]: Finds regions of enrichment from spatially assigned *P* values
- *Probe Lasso*[27]: A method that offsets CpG density bias via moderating the candidate window in which DMRs are defined against the local density of their constituent CpG sites. *Probe Lasso* forms part of the *ChAMP* package in Bioconductor. Despite both occurring in the field of high-dimensional data, this method is unrelated to LASSO feature selection [50].

Our justification for the use of *limma* is that it shrinks sample variances towards a pooled estimate, giving more stable results when the sample size is small [43], as is the case with our test data. For the second criterion, since the WGBS DMR-finding method itself retains all candidate CpG sites regardless of their accompanying annotation (or lack of), we used only those 450K DMR-finding methods that do the same. It should be noted that although *Probe Lasso* arranges probes into candidate DMRs by their annotation prior to DMR finding, we have included it in our testing both because it does so exhaustively (that is, including those probes without a gene or CpG-island association).

All reasonable efforts were made to standardise each method towards a parametric parity, while staying as close to their defaults as possible. Crucially, the definition of a minimum of 1,000 nucleotides for DMR separation was passed to the appropriate argument of each implementation. Each method is able to return a ranked list of DMRs. Various metrics describing the parameter specifications and computational speed of each candidate method can be found in Table 1.

**Table 1:** **Usage of the four candidate methods in this study**

Method	Implementationlanguage	Relevant parameters used in call ^a^	Computational time (to nearest second) ^b^
*DMRcate* v1.2.0	R	lambda = 1000, C=2, p.adjust.method = ~BH~, pcutoff = *α*	129
*Bumphunter* from *minfi* v1.10.2	R	cluster = NULL, maxGap = 1000, smooth = TRUE, smoothFunction = loessByCluster, cutoff = *α*	58
*comb-p* v0.35	Python	–dist 1000 –seed *α*	96
*Probe Lasso* from *ChAMP* v1.2.7	R	filterXY = F, mafPol.lower = 0, mafPol.upper = 1, lassoRadius = 1000, minDmrSep = 1000, adjPVal = 1, minSigProbesLasso = 1, DMRpval = *α*	6,867

^a^Argument *α* indicates the tuned parameter for which sets of called DMRs were produced. Any omissions imply function defaults. ^b^Computational time is for the VA vs SA comparison, which is the time needed to fit the whole 450K methylome (using parameters in the previous column) with a single core of a clean Dual Xeon eight-core E5-2650 compute node, using 64 GB of virtual memory.

Each method takes a slightly different approach to multiple testing. *DMRcate* performs a BH correction on the *P* values corresponding to all points at which the *χ*^2^ statistic is calculated, and takes the minimum adjusted *P* value in the region as representative, since all CpG sites within the specified window contribute to the support at that point. Very similarly, the *P* values from *Bumphunter* regions are the minimum probe FDRs at which the associated area may be called significant, but Storey’s optimal discovery procedure [51] is used as a correction routine instead. *comb-p* uses a one-step Šidák correction [52] on the estimated region-wise Stouffer–Liptak *P* value. Very similarly to *comb-p*, *Probe Lasso* assigns *P* values to each of the ‘lassoed’ regions via Stouffer’s method [53], and then performs a region-wise BH correction. Hence, to avoid confounding due to unstandardised multiple testing procedures, we used quantile thresholding, commensurate with genomic coverage of DMRs called, for each validation point on the tuning domain. This was done by titrating values of the tuning parameter *α* (Table 1) appropriately to ensure complete coverage along the recall domain for each validation curve drawn. We generated 200 GRanges [54] objects from the resultant lists of DMRs for the 200 values of *α*, each corresponding to a point on the precision–recall curve.

### Performance measure: area under the precision–recall curve

We chose to represent the performance of each candidate method via the area under the precision–recall curve (AUCPR), since receiver operating curves are susceptible to class skew [55, 56]. In studies such as this, where an overwhelming proportion of the genome is evaluated as true negative in the confusion matrix, precision–recall statistics can be a preferred option for genomic region prediction [57, 58].

**Figure 3:**
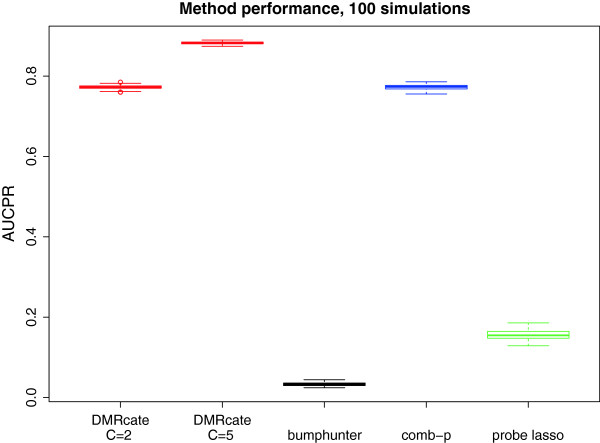
**Box plots of AUCPR representing method performances for 100 simulations of 450K data.** AUCPR, area under the precision–recall curve.

For the 100 simulations, overlaps with ground truth DMRs were calculated across *α* for each candidate method at a nucleotide resolution using GRanges objects, and the resulting precision–recall curves were drawn. The performance of each candidate method is shown in Figure 3. *DMRcate*’s performance is comparable to *comb-p*’s for its default option; however, this is improved by increasing the *C* parameter (see Methods), which shrinks the kernel size. This is because *DMRcate* incurs false positives in regions flanking the ground truth when the kernel is too large, due to the support extending past ground truth bookends.

**Figure 4:**
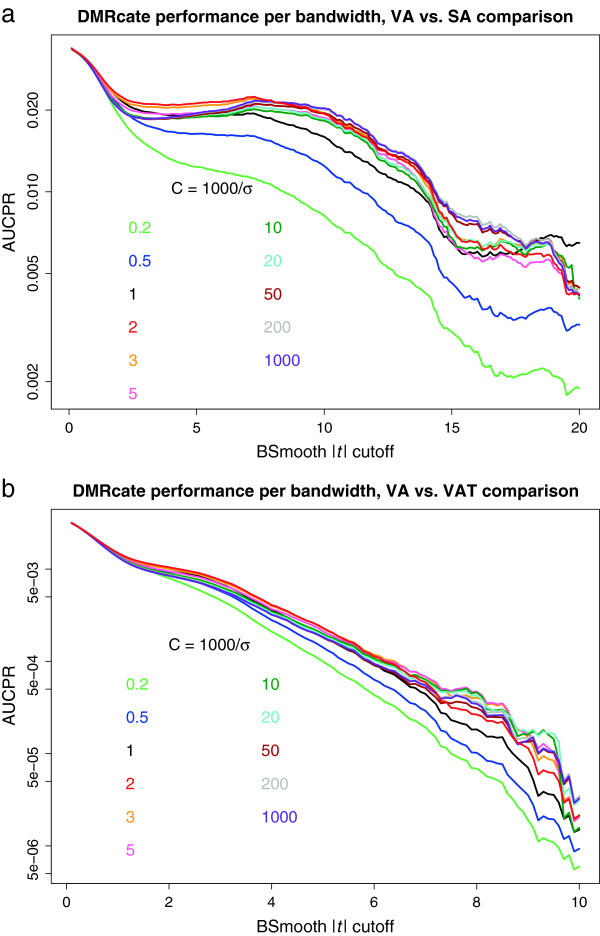
***DMRcate*** **bandwidth validation.** **(a)** AUCPR (log scale) for different values of the *DMRcate* bandwidth, validated on WGBS DMRs from the VA vs SA comparison. **(b)** The same plot for the VA vs VAT comparison. AUCPR, area under the precision–recall curve; DMR, differentially methylated region; SA, subcutaneous adipocyte; VA, visceral adipocyte; VAT, visceral adipose tissue; WGBS, whole-genome bisulfite sequencing.

However, when validating on real (WGBS) data, we needed an estimate of the optimal kernel size, due to the greater number of CpGs interrogated by sequencing. A parameter value of *C*=2 (kernel size = 500 bp) was found to be near optimal for both the VA vs SA and VA vs VAT comparisons, for moderately sized *BSmooth* |*t*| cutoffs (red series, Figure 4a,b). Kernel size (*σ*) seems to matter little when large areas of the methylome are called as ground truth (low *BSmooth* |*t*| cutoff), a *σ* value of approximately 500 bp (*C*=2, see Methods) is optimal for modest cutoffs, but for high-stringency cutoffs, very small kernels are optimal. If the DMRs called are too large (such as when *C*=0.2 and *σ*=5,000), then their poor precision will penalise overall performance.

**Figure 5:**
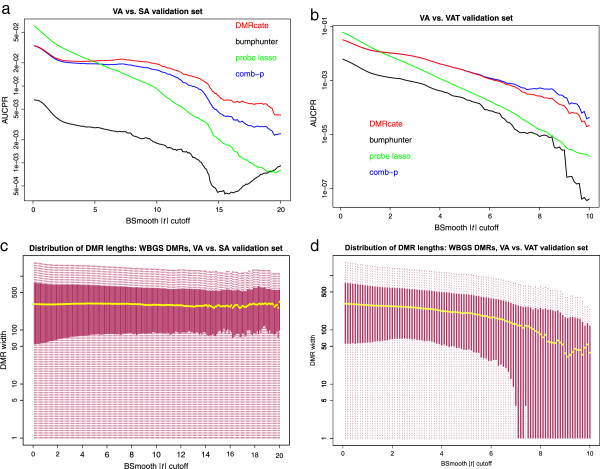
**DMR caller candidate performance and accompanying ground truth DMR size.** **(a)** AUCPR for four 450K-DMR-calling methods (log scale), validated on a series of DMRs called from WGBS from the same DNA samples, called by varying the *BSmooth* |*t*| cutoff parameter, for the VA vs SA comparison. (**b)** AUCPR (log scale) on the candidate methods for the VA vs VAT comparison. **(c)** *BSmooth*-called DMR widths (log scale) from the WGBS data, for the VA vs SA comparison. Yellow dots indicate the median length in this and the following panel. **(d)** *BSmooth*-called DMR widths (log scale) from the WGBS data, for the VA vs VAT comparison. AUCPR, area under the precision–recall curve; DMR, differentially methylated region; SA, subcutaneous adipocyte; VA, visceral adipocyte; VAT, visceral adipose tissue; WGBS, whole-genome bisulfite sequencing.

*DMRcate* clearly makes the best DMR predictions for the VA vs SA comparison for approximately the top 75% of the *BSmooth* |*t*| cutoff domain (Figure 5a). For the VA vs VAT comparison (Figure 5b), *DMRcate* and *comb-p* show near-equal performance for the lower half of this domain, with *comb-p* outperforming the other methods as DMR lengths attenuate (Figure 5b,d).

Despite accepting all candidate CpG sites for finding DMRs, as per our previously stated criterion, even when cutoff = 0 is specified, *Bumphunter* only calls DMRs comprising a subset of the original corpus of CpG sites, for all validations performed. Seemingly, *Bumphunter* internally filters out regions where the CpG site density is too sparse. In fact, for both data sets, fewer than 20% of the original candidate CpG sites (92,524/466,190 and 92,505/466,263 probes for the VA vs SA and VA vs VAT data sets, respectively) are retained as constituents of DMRs. Similarly low representations were observed for the simulated data sets. Hence, its performance is greatly affected by false negatives.

By contrast, *Probe Lasso* is more sensitive to the WGBS-derived DMRs than the other three methods (see Additional file 1: Figure S4), but its performance is chiefly hindered by false positives (for specific examples see Additional file 3: Table S2).

### Computational time

The version of *Bumphunter* used in this study (Table 1) is the best performing method in terms of computational time, being able to fit a whole 450K methylome in serial on a Xeon eight-core E5-2650 (CSIRO GPU cluster, Top500 rank 180, June 2014) in 58 seconds, followed by *comb-p* at 96 seconds and *DMRcate* in just over 2 minutes. *Probe Lasso* took considerably longer to call DMRs, needing approximately 114 minutes. This is largely the result of method standardisation and maintaining parametric parity. When the parameters *adjPVal* and *minSigProbesLasso* are decreased and increased respectively, the time *Probe Lasso* requires to call DMRs shortens drastically and becomes comparable to the other three methods.

## Discussion

### Analysis of results

The performance profiles of each method are partly influenced by the extent of genomic coverage its DMRs call. Intuitively, since the 450K array has a reduced representation, DMRs called from it that have flanking regions may infer nearby true DM, detectable by WGBS, where the 450K probe coverage is sparse. *Probe Lasso*’s increased sensitivity, and its superior performance for predicting low-stringency DMRs, are because its DMR bookends are defined not by CpG sites themselves, but the size of the lasso encompassing them. Hence, the flanks of these DMRs can predict nearby sequencing-derived DMRs.

As the sequencing-derived DMR definition becomes more stringent, *Probe Lasso*’s precision begins to drop away more quickly than the other methods. This is likely because the 450K-derived DMR flanks become a liability, reporting false positives, as the sequencing-derived DMRs become sparser. As described earlier, this phenomenon is more pronounced in the VA vs VAT comparison (Figure 5b), likely because of the smaller biological effect. Since the effect shift is more subtle, then the DMRs called from the WGBS assays are shorter than those called from the VA vs SA comparison, and hence the precision drops more quickly for generously flanked DMR estimates. In addition, *Probe Lasso*’s DMR definitions are tethered to the annotation of their constituent CpGs, in that they divide genomic regions along both the CpG-island-relation axis (e.g. shore, shelf and island) and the gene-relation axis (e.g. promoter, gene body and 5^′^ UTR). True DMRs that straddle multiple annotations are split and called separately, likely penalising the sensitivity of the method as well.

As mentioned earlier, *Bumphunter* does not call DMRs where the CpG coverage is sparse. This is possibly because the smoothing function (loess) is shift-invariant, and hence estimation of DM at CpG interstices in these regions is unreliable. In contrast, the degrees of freedom *b* (see Methods) on which *DMRcate*’s *χ*^2^ statistic is calculated simply tends to 1 when there are few or no methylation sites to be fitted, giving less power. Regardless, *DMRcate* still arranges every candidate CpG into a DMR, even if it is a singleton (as do *Probe Lasso* and *comb-p*).

In addition, *DMRcate* uses unsigned weights (*limma*’s *t*^2^s) to pass to the kernel estimator, whereas *Bumphunter* retains the sign of the DM for smoothing. This retention may result in a loss of DM sensitivity, due to signal cancelling, where the direction of effect changes abruptly, as described in Day *et al.*[59]. *comb-p*’s and *Probe Lasso*’s DMRs are exempt from this complication, since they are derived from the unsigned individual CpG site *P* values themselves. Even though the *BSmooth* algorithm takes a similar approach to *Bumphunter*, in that it smooths signed methylation values, the finer granularity of sequencing data and the subsequent smaller running mean of the smoothing engine (101 nucleotides) means that it adjusts much more quickly to these abrupt sign changes. It is possible that even in the regions where the 450K probe density is high enough for *Bumphunter* to call DMRs, often they are still not dense enough to sensitise *Bumphunter* to the crossovers quickly enough, compared to the other three methods. From a biological perspective, given that promoter methylation is associated with silencing, and genic methylation is involved in upregulation (see Background), it follows that a region of short-range methylation sign change is of high interest for the epigenetic control of gene regulation. Example DMR-calling patterns associated with the HOXC4 locus from all four candidate methods (plus *BSmooth*) from the VA vs SA comparison are shown in Additional file 1: Figure S5.

*comb-p*’s performance seems to be highly contingent on the degree of effect shown in the comparison tested. For the subtle effect seen in VA vs VAT, it is able to predict DMRs with a high degree of precision, essentially matching *DMRcate*’s performance for lower-stringency DMRs (Figure 5b). The pattern of DMRs found by both in this instance is likely very congruous, given the similar amount of genomic coverage of their DMRs (see Additional file 1: Figure S4), that they both define DMR bookends by CpG sites, and that they both use a peak-hunting heuristic. The very similar performance profiles also suggest an upper limit to which DMRs can be detected in data from reduced representation platforms, when the biological effect is small. *comb-p*’s relative performance improves as the median DMR length starts to decrease rapidly after the *BSmooth* |*t*| cutoff rises above 6 (Figure 5b,d). At this point, *DMRcate* likely incurs more false positives due to kernel support inferring false DM beyond the flanks of these very small true DMRs, as seen with the simulated data. However, this effect can be minimised by shrinking the kernel size (Figure 4b).

Conversely, for a comparison with a larger effect (VA vs SA), the WGBS-derived DMR length distribution remains relatively constant throughout the cutoff domain (Figure 5c), reflecting real biological differences even at high values of the |*t*| threshold. With these DMRs, *comb-p*’s predictions are not as compatible as *DMRcate*’s are, and hence are more susceptible to both false positives and false negatives at the given *BSmooth* |*t*| cutoff (Figure 5a). Thus *DMRcate* is relatively robust to biological effect size. We would recommend *comb-p* for DMR finding in cases where the effect size is very small – such as, for example, where no differential probes with *P*<0.05 are returned by *limma* after BH correction – and if *DMRcate*’s output is unsatisfactory. In addition, *DMRcate* has the following practical advantages over *comb-p*:

- A tunable kernel size parameter for optimum performance
- Readily annotated results for each list of DMRs
- R implementation, facilitating data integration pipelines with other Bioconductor tools

### Future directions

The *DMRcate* method template of modelling the local statistic *Y*_*i*_ as a scaled *χ*^2^ random variable is versatile enough to extend into distributions other than *t*^2^. For example, Cox proportional hazard coefficients can be modelled as such [60], given appropriate specifications. For a general linear model with *d* parameters, we can test the significance of the model overall by specifying *μ*=*d*−1 and *Y*_*i*_=*F*_*i*_, where *F*_*i*_ is the robust *limmaF* statistic (see Table 2). For the variability option, we can also introduce a *k*-level blocking factor, to measure within-group variation across the sample group. In this scenario, *V*_*i*_ would be calculated for each CpG site as the sample within-block variance, with the *F* statistic degrees of freedom being (*n*−*k*,*∞*).

**Table 2:** **Analysis options and local test statistics**

Analysis	***Y***_***i***_	***μ***	***ν***
Two groups (DMRs)	*Limma*	1	*ν* ^⋆^
Contrast (DMRs)	*Limma* contrast	1	*ν* ^⋆^
Variability (VMRs)	*V*_*i*_/*V*	*n*−*k*	*∞*

DMR, differentially methylated region; VMR, variably methylated region.

*DMRcate*’s implementation also has scope for expansion into fitting data from platforms beyond the 450K array. With the proliferation and decreasing cost of next-generation sequencing technology and data generation [61–63], fitting WGBS data is a likely future application. This will need both greater code flexibility with regards to annotation and, most likely, parallelisation incorporated into the implementation to cope with the large amount of data input. The method need not be restricted to methylation data either, nor human; any data that seeks to identify local genomic regions by fitting points spatially, for example SNP data [64], is a candidate for this type of modelling. As the wealth of available genomic data grows exponentially, methods that reduce, generalise and interpret data are in greater demand than ever, and as such we see prolific application of this form of genomic inference in the future.

## Conclusions

*DMRcate* calls DMRs derived from replicated 450K samples in a competitive manner compared to equivalent implementations in the field, both in terms of prediction and computational time. For the methods tested, *DMRcate* and *comb-p* are the clear frontrunners in terms of predictive performance. This has been validated on WGBS assays performed on the same DNA samples. The definition of region bookends appears to be critical to accurate prediction of DM. In addition, non-agnostic approaches to either the direction of effect or genomic annotation (such as CpG-island association) of methylation sites appear to hinder the detection of DMRs. As such, the role of methylation in humans is likely more diverse and more complex than current hypotheses derived from 450K analyses attest, and the approach of our method can serve as a valuable tool in elucidating this complexity.

## Methods

Our approach to determining DMRs or other genomic regions of interest has the following steps:

- According to the analysis option chosen, calculate variances or apply standard linear modelling to the data using treatment labels and any other relevant clinical data and covariates.
- Apply Gaussian smoothing to the resulting per-CpG-site test statistics using a given bandwidth, *λ*.
- Model the smoothed test statistics using the method of Satterthwaite [65].
- Compute *P* values based on this model.
- Apply standard *P* value adjustment.
- Use a threshold on adjusted *P* values to give FDR-corrected significant CpG sites.
- Agglomerate nearby significant CpG sites, again using *λ*.

The agglomeration bandwidth, *λ*, is supplied by the user. We use a default value of *λ*=1,000 bp, as do *Bumphunter* and *Probe Lasso*.

### Linear modelling: local *F*statistics

Our method has three different analysis options. These correspond to the rows of Table 2. The first two options begin with standard linear modelling using *limma*[43], which uses selected factors and covariates, and variance shrinkage, to fit a linear model to all CpG sites in parallel. The first and default option is applicable to the simplest design where there are two treatments. In this analysis, *limma* produces a *t* value, *t*_*i*_, at each CpG site. This is a signed statistic for assessing the difference between the treatment effects at this CpG site. A key strategic decision in the design of our method is, in contrast to, say, Bumphunter [25], to combine genomically nearby CpG site effects *without regard to direction of effect*. This is because local hypermethylation in one treatment relative to others may be followed immediately in the genome by coordinate hypomethylation as part of the same regulatory mechanism [59]. We therefore use  as our local statistic. This is an *F* statistic with a single degree of freedom in its numerator. The denominator degrees of freedom, *ν*^⋆^, is estimated by *limma*; this is the degrees of freedom of the shrunk per-CpG-site variance estimate, where the shrinkage factor is determined by an empirical Bayes process. This shrinkage is usually quite strong, in which case *ν* is relatively large.

The second analysis option is, in a statistical sense, very similar. Here a more complex linear model is used, with possibly many treatments and/or other factors, but we specify a particular contrast that we want to test. Again the output at each CpG site is a *t* value and we use  which, as in the first option, is an *F* statistic with (1,*ν*^⋆^) degrees of freedom.

The third option is simpler and does not use *limma*. Here we are interested in genomic regions that are highly variable. By default, this option computes the variance, *V*_*i*_, of *M* values across the *n* samples. Under the null assumption that all CpG sites have the same variance, *τ*^2^, each *V*_*i*_ is distributed as:


We now let *V* be the mean of all the *V*_*i*_s and set:


As there are over 450,000 CpG sites, *i*, *V* is a very precise estimate of the supposed common variance, *τ*^2^, so *Y*_*i*_ is very close in its distribution to , which is equivalent to an *F* statistic with (*n*−1,*∞*) degrees of freedom.

### Kernel smoothing

We now smooth the statistics *Y*_*i*_ at the locations *x*_*i*_ with a Gaussian smoother. This and the other steps below are done separately for each chromosome. Let *x*_1_<⋯<*x*_*n*_ be the CpG sites for the current chromosome.

Gaussian kernel weights are defined as:


The kernel scale factor *σ* should be proportional to the bandwidth *λ*. For example, in the experiment shown in Figure 4a,b, we set *σ*=*λ*/*C* and compared the performance at different values of *C*.

We then use an efficient sparse computation to produce the following three sums at each CpG site *x*_*i*_ in (*x*_1_,…,*x*_*n*_):


### Model for smoothed data

The second and third of these sums, *S*_*K*_(*i*) and *S*_*KK*_(*i*), depend only on the CpG sites, {*x*_*i*_}, not the statistics, {*Y*_*i*_}. These are, from a statistical point of view, constants. They are needed, however, as described below. The first term, *S*_*KY*_(*i*), is the kernel-weighted local model fit statistic. Each *Y*_*i*_ is assumed to be an *F* statistic with (*μ*,*ν*) degrees of freedom where *μ* and *ν* are known values (see Table 2). We now model the distribution of *S*_*KY*_(*i*).

In the context of density estimation, Duong [66] uses the central limit theorem to show that the equivalent of *S*_*KY*_(*i*) is approximately normal in its distribution if the number of points in the vicinity of *x*_*i*_ (in this case, within about 1,000 bp of *x*_*i*_) is large. This approximation will be poor in our case for the many CpG sites that have few nearby CpG sites, or none. On the other hand, explicit modelling of *S*_*KY*_(*i*) as a linear combination of *F* random variables would be exact, but mathematically complex. We take an intermediate approach in which we assume *ν* is large so that the denominator in each *F* statistic is close to one. This implies that:


This is exactly true for the variability option and approximately true for the other *limma*-based options where *ν*=*ν*^⋆^, which is generally large due to variance shrinkage.

With this assumption we use the approximation of Satterthwaite [65], in which we model *S*_*KY*_(1) by a scaled chi-squared random variable, , where the constants *a*_*i*_ and *b*_*i*_ are chosen to match the first two moments. (See also Buckley and Eagleson [67] for an alternative approach).

The mean and variance of  are *a*_*i*_*b*_*i*_ and  respectively, while if :


Now *a*_*i*_ and *b*_*i*_ are defined by the moment matching equations:


and solving these leads to:


### *P*values and differentially methylated region segmentation

**Figure 6:**
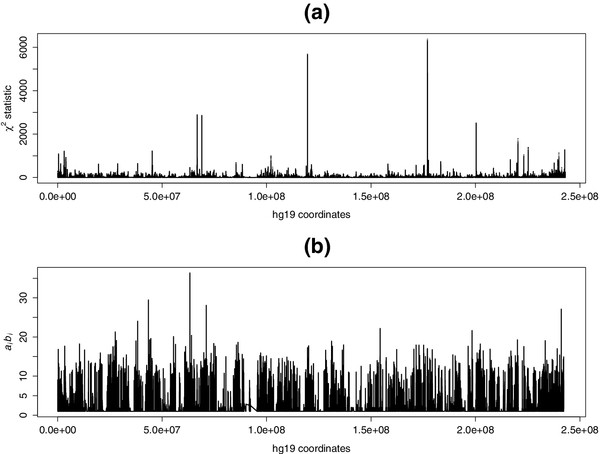
**Observed and expected kernel function values.** **(a)** *χ* ^2^ statistic  for all probes from the VA vs SA comparison fitted to chromosome 2. **(b)** Expected value *E* *S*_*KY*_ for the same domain. SA, subcutaneous adipocyte; VA, visceral adipocyte.

We can now compute a *P* value, *P*_*i*_, for each location *x*_*i*_ by comparing:


to a *χ*^2^ distribution with *b*_*i*_ degrees of freedom. An example of the *χ*^2^ value from chromosome 2 of our VA vs SA comparison, compared with the expected value , can be seen in Figure 6.

We then perform a BH correction [68] on the *P* values *P*_*i*_, giving adjusted *P* values, *Q*_*i*_, and retain the subset of CpG sites *x*_*i*_ where *Q*_*i*_ is smaller than a given threshold (usually 0.05). Finally, regions (DMRs or VMRs, depending on the analysis option) are defined by collapsing groups of the remaining CpG sites that are at most *λ* nucleotides from each other.

### Implementation

*DMRcate* is the R package implementation of this method, and is available from the Bioconductor repository [69]. All the user needs to provide to the *DMRcate* workflow is an Illumina probe ID-indexed matrix of methylation measurements and, for DMR finding, a model matrix (optionally with an additional contrast matrix) reflecting the experimental design. *DMRcate*’s experimental design idiom is lifted wholesale from *limma*, hence it can fit any given contrast from a *limma* model. The workflow assumes that the user has already normalised the data according to their preferred method, and removed bad-quality probes via detection *P* values and low bead count. As a further preprocessing option, *DMRcate* provides an optional filtering function, removing probes whose reported methylation level may be confounded by SNPs, and/or by cross-hybridisation [45]. Further investigation of a 450K data set used in this study reveals that SNPs within two nucleotides of the target CpG site have a perturbed beta distribution (Additional file 1: Figure S1). If the samples are from mixed-sex groups, the option of removal of probes hybridising to X and Y chromosomes is also provided.

Initial output consists of a data frame describing each region, ranked by its corresponding *P* value. Useful information such as genomic coordinates, gene associations and number of constituent CpGs per region are also reported. The user may specify any positive bandwidth they like. Longer bandwidths allow for interrogation on a broader, even chromosomal, scale, while shorter bandwidths potentially allow identification of focal regions of DM. Post-fitting, the user has the option of filtering out any region that does not have at least one constituent CpG site with a beta fold change greater than a specified threshold. As an alternative to using genomic coordinates, *DMRcate* has a consecutive option that assumes all assayed CpGs are equally spaced. Wrappers for GenomicRanges object and whole-genome BedGraph production are provided. For visualisation, a separate plotting function for individual DMRs is also provided. An example of the graphical output of *DMRcate* can be found in the online vignette, and as part of Additional file 1: Figure S5. A complete description of *DMRcate*’s functionality and user options is available from the manual [70].

## Electronic supplementary material

Additional file 1: **Supplementary material.** (PDF 296 KB)

Additional file 2: **Table S1.** GO Biological Process terms, ranked by FDR, obtained from *goseq* for tissue-specific DMRs from Slieker *et al.* for blood, buccal cells, liver, muscle, pancreas and spleen. Gene associations (if any) from the top 150 DMRs, as found by *DMRcate*, were denoted as differentially expressed when calling *goseq* and can be found in the final column of each sheet. Significantly enriched terms are highlighted in green. (XLSX 4230 KB) DMR, differentially methylated region; FDR, false discovery rate; GO, gene ontology. (XLSX 4 MB)

Additional file 3: **Table S2.** True positives, false negatives and false positives (in nucleotides) returned by *DMRcate*, *Bumphunter*, *comb-p* and *Probe Lasso* for validations on simulated DMRs (Sheet 1), *BSmooth* calls on the VA vs SA comparison at *t* cutoff = 10 (Sheet 2), and on the VA vs VAT comparison at *t* cutoff = 5 (Sheet 3). (XLSX 70.4 KB) DMR, differentially methylated region; SA, subcutaneous adipocyte; VA, visceral adipocyte; VAT, visceral adipose tissue. (XLSX 70 KB)

## Abbreviations

AUCPR: Area under the precision–recall curve
BH: Benjamini–Hochberg
bp: base pair
DM: Differential methylation
DMR: Differentially methylated region
FDR: False discovery rate
GO: BP: Gene ontology terms for biological process
kb: kilobase pair
MDS: Multidimensional scaling
SA: Subcutaneous adipocyte
SNP: Single nucleotide polymorphism
VA: Visceral adipocyte
VAT: Visceral adipose tissue
VMR: Variably methylated region
WGBS: Whole-genome bisulfite sequencing.
