# Supplementary material for: De novo identification of differentially methylated regions in the human genome
Source: Epigenetics Chromatin. 2015 Jan 27;8:6. doi: 10.1186/1756-8935-8-6 (PMC4429355; doi:10.1186/1756-8935-8-6)
Supplement: Supplementary file 1 — Additional file 1: Supplementary material. (PDF 296 KB) [file 13072_2014_357_MOESM1_ESM.pdf]

Supplementary Material: *De novo* identification of differentially methylated regions in the human genome

Description of Simulated Data

1. Base information

   We began by constructing some empirical methylated structure on the human genome, as represented by the 485,512 probes on the 450K array.

   (a) Unmethylated and fully methylated probes

   All probes were classified as fully methylated or unmethylated according to the values of an array taken from a sample of human leukocytes. Probes with normalised methylation score (beta) greater than 0.5 were classified as fully methylated, others as unmethylated. This is a fair approximation of reality, as beta value distributions are strongly bimodal with modes near 0 and near 1. The proportions of unmethylated and fully methylated probes were 44.5 and 55.5% respectively.

   (b) Candidate DMRs based on promoter regions

   Candidate DMRs were defined as genomic regions encapsulated by runs of probes annotated as "TSS200" or "TSS1500" at most 1000 nucleotides apart, each of which we used in the simulation as a candidate DMR. There were 21,617 of these candidate DMR regions, constituted of between 1 and 88 probes. The median number of probes was 6, the mean 6.5, and in total the candidate DMRs made up 28.8% of the probes on the array.

2. Randomised location and methylation levels of DMRs

   In each simulated data set, 5% of the 21,617 candidate DMRs were selected as being up (hypermethylated) in the treatment samples, 5% as being down (hypomethylated) in the treatment samples, and 90% as having no signal. As 5% of 21,617 is 1080.9, this process amounts to choosing 1081 of the candidate DMRs as 'up' regions and 1081 as 'down' regions.

   We determined that the true methylation difference at all DMRs would be exactly 0.2. For each region we produced two beta levels:

   beta1 $\sim$ Uniform(0.01, 0.79)

and

beta2 = beta1 + 0.20

This means that

beta2 ~ Uniform(0.21, 0.99)

For "up" regions, the base methylation level for the control samples was set as beta1, and for treatment samples as beta2. For "down" regions this allocation was reversed.

3. Structure of simulated data sets

   We simulated 10 replicate samples in each of the two sample groups, control and treatment. Thus each simulated data set was generated as a 2D array with 485,512 rows and 20 columns. The first 10 columns were control samples, the last 10 columns wer treatment samples.

4. Simulation of beta values: Inside DMRs

   First we simulate beta values in each of the DMRs selected in stage (2) above, using the two beta level values, one for control samples and one for treatment samples. Random values are generated for all samples and all probes in the region. A beta distribution is used to generate random data (function `rbeta()` in R). The beta distribution has two parameters, a and b. The mode of the beta(a, b) distribution is

   mode = (a - 1) / (a + b - 2)

   We choose parameters a and b so that this mode is equal to the specified beta 'level' and so that

   a + b + 2 = K = 100

   This value was chosen to give a realistic amount of variability in the sampling distribution. The following R code implements this simulation, given K and 'level':

```
mu <- mode
r <- mu/(1 - mu)
B <- K/(1+r)
A <- r*B
a <- A + 1
b <- B + 1
beta <- rbeta(a=a, b=b)
```

5. Simulation of beta values: Outside DMRs

   For the remaining probes (i.e. all those outside the selected DMRs) beta values were simulated from two distributions, according to the binary unmethylated/fully-methylated status of the probe (see step 1). For unmethylated probes we sample from

   `rbeta(a = 2.4, b = 20)` (Mode = 0.07)

   and for fully methylated probes we sample from

   `rbeta(a = 14, b = 3)` (Mode = 0.87)

These distributions were chosen to match sample beta data for the bulk of CpG sites.

6. Final Adjustment

   To avoid beta values very close to 0 and 1, transform beta as

   beta = (beta + delta)/(1 + 2*delta)

   where delta = 0.01.

7. Replicate simulations

   The entire simulation was repeated 100 times.

Supplementary Figures

Supplementary Figure 1: Beta distribution of 450K probes from visceral and subcutaneous adipocytes, and visceral adipose tissue (9 samples total) by their proximity to a SNP. "All SNP probes" refers to the 153,113 probes listed by Illumina whose values may potentially be confounded by a SNP.

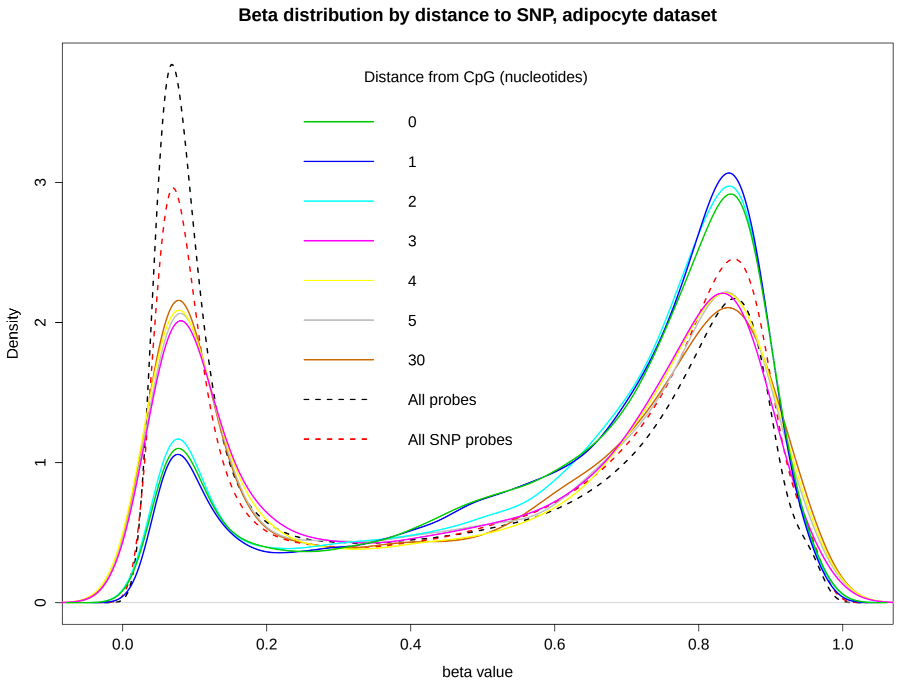

Supplementary Figure 2: Coverage curves, over the 28,217,448 human genome (build hg19) CpG sites, of the nine DNA samples used in this study.

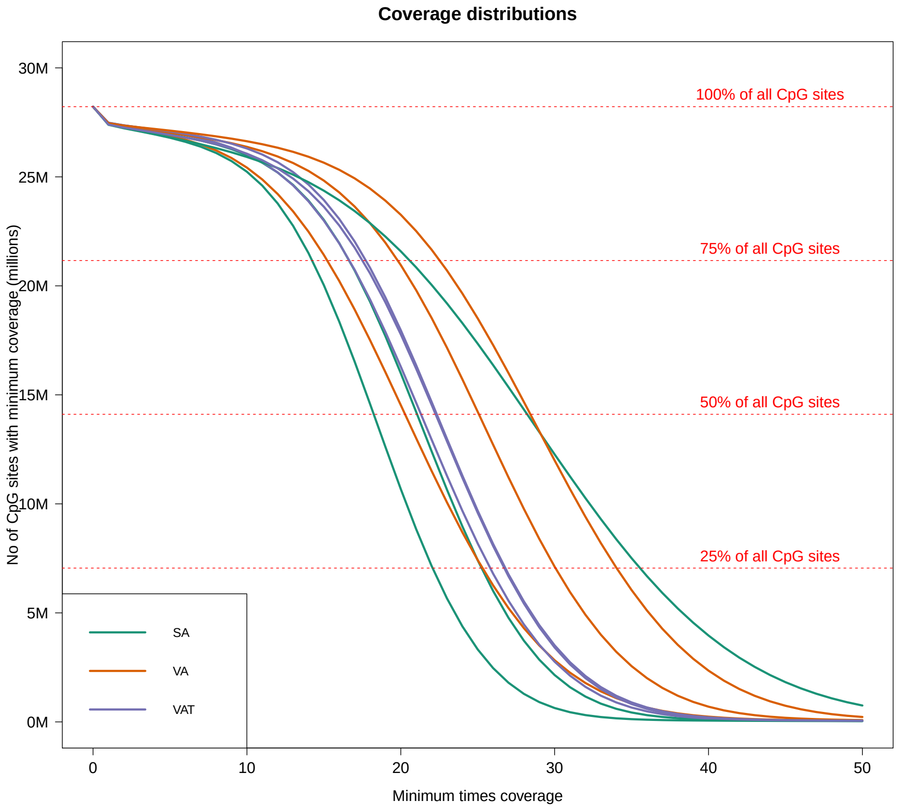

Supplementary Figure 3: Proportions of CpG sites from the 450K array, as constituents of VA vs. SA DMRs, by (a) CpG Island annotation, called by *DMRcate* from 450K data, (b) CpG island annotation, called by *BSmooth* from WGBS data, (c) gene annotation, called by *DMRcate* from 450K data and (d) gene annotation, called by *BSmooth* from WGBS data. X-axes in (a) and (c) are reversed and log-scaled. Red and blue horizontal lines indicate the proportion of the 450K array represented by open sea and non-gene-associated probes.

(a)

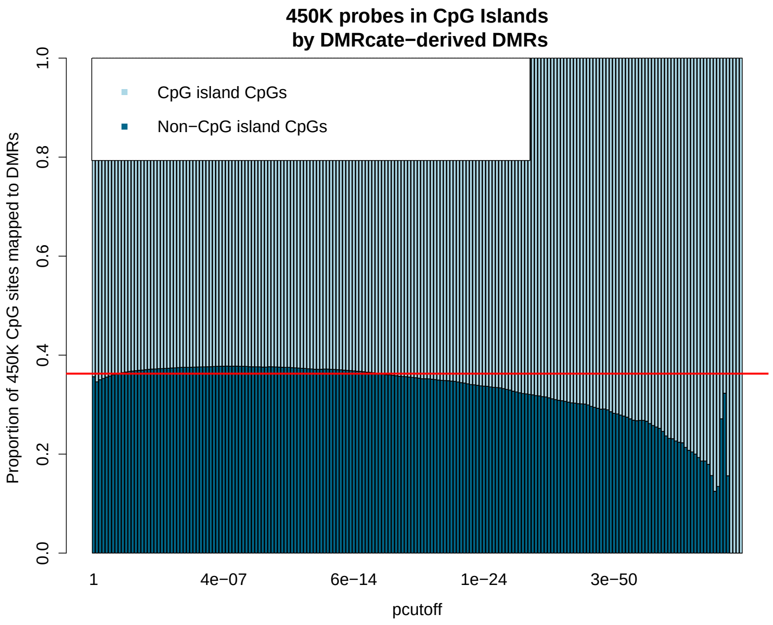

(b)

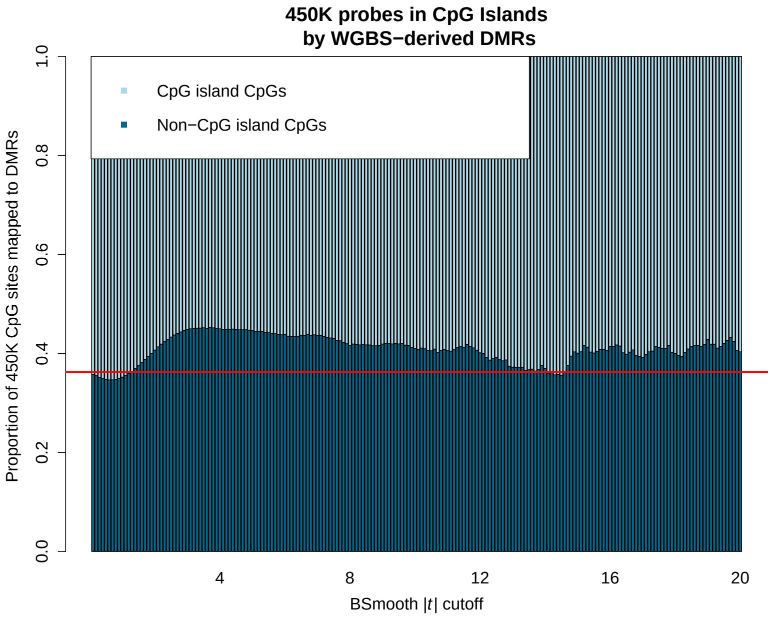

(c)

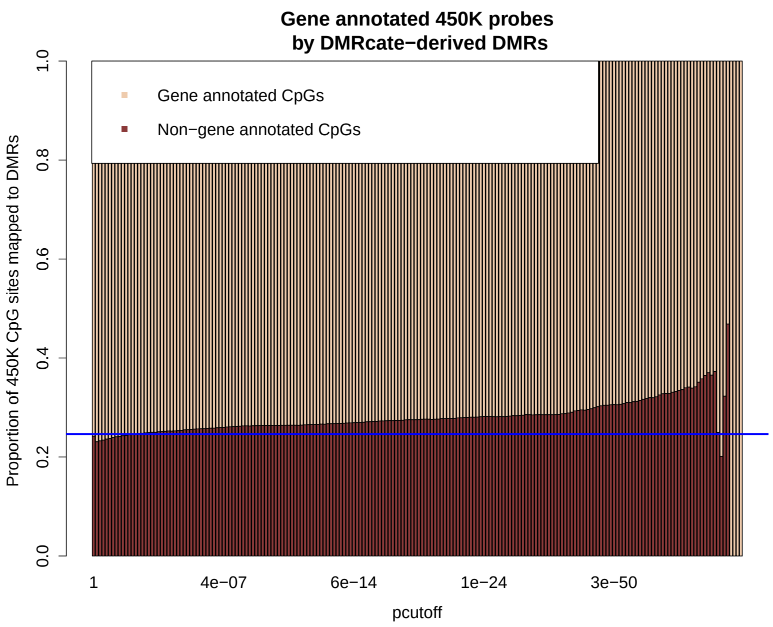

Gene annotated 450K probes
by DMRcate−derived DMRs
Gene annotated CpGs
Non−gene annotated CpGs
Proportion of 450K CpG sites mapped to DMRs
0.0
0.2
0.4
0.6
0.8
1.0
1
4e−07
6e−14
1e−24
3e−50
pcutoff

(d)

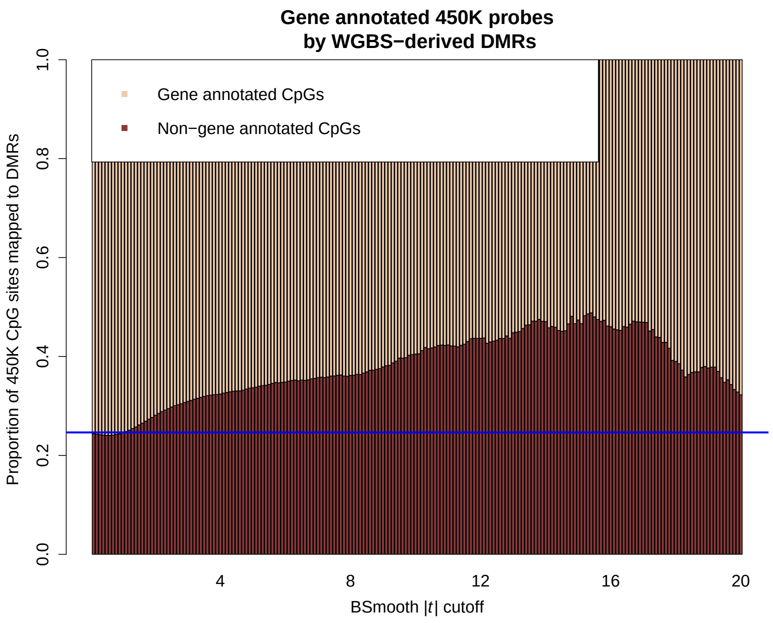

Gene annotated 450K probes
by WGBS−derived DMRs
Gene annotated CpGs
Non−gene annotated CpGs
Proportion of 450K CpG sites mapped to DMRs
0.0
0.2
0.4
0.6
0.8
1.0
4
8
12
16
20
BSmooth |t| cutoff

Supplementary Figure 4: Maximum possible sensitivity of the tuning parameter domain of 450K-derived DMRs from the four candidate methods against *BSmooth* $|t|$ cutoff for WGBS-derived DMRs, for the VA vs. SA comparison. *DMRcate*'s and *comb-p*'s profiles are identical since they both define DMR bookends in the same way.

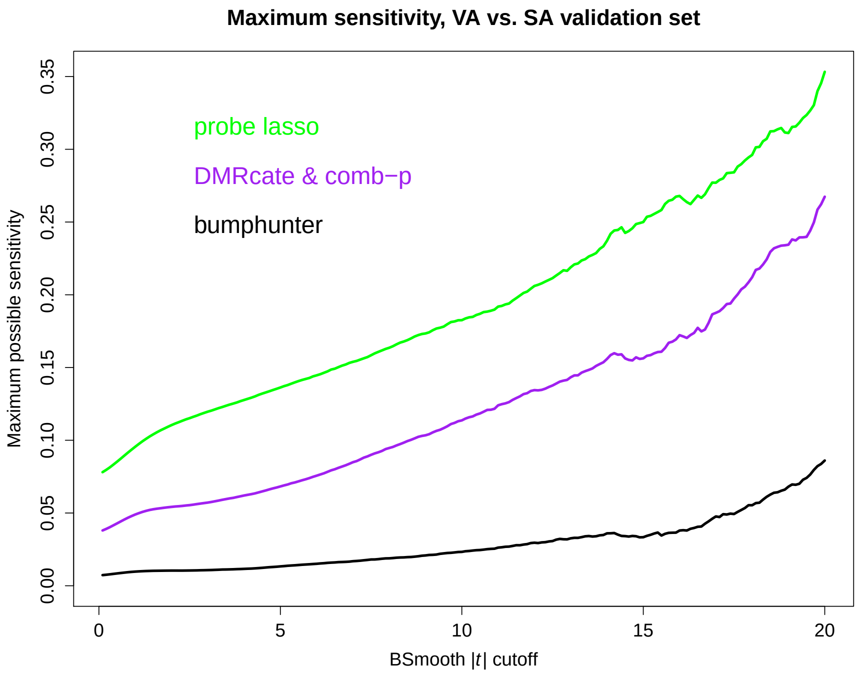

Supplementary Figure 5: A section of the HOXC4 locus for which DMRs have been called from the VA vs. SA comparison. The top half of each plot constitutes an output from the *DMRcate* package function DMR.plot() showing actual 450K beta values from visceral (purple) and subcutaneous (orange) adipocytes, with joined lines indicating median methylation. Annotation in coloured squares along the top is as per the *DMRcate* manual. The bottom half of each plot shows where DMRs have been called by sequencing (maroon), and then from the 450K data from the four candidate methods. Right hand side axis indicates either the thresholds or their corresponding quantile of total 450K CpG sites that were returned as DMR constituents, for which DMRs were called.

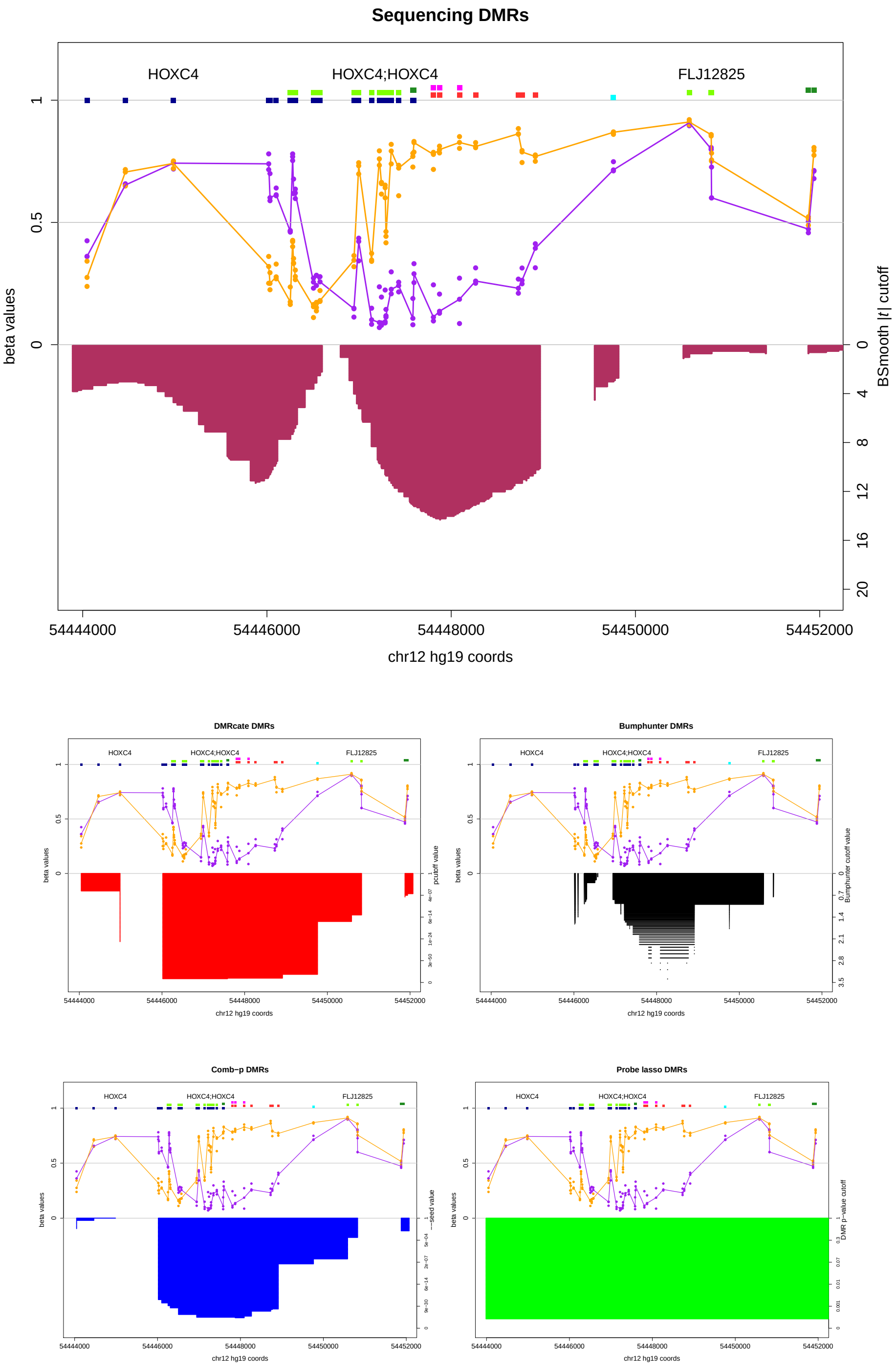
Sequencing DMRs
HOXC4
HOXC4;HOXC4
FLJ12825
beta values
BSmooth |t| cutoff
54444000
54446000
54448000
54450000
54452000
chr12 hg19 coords
DMRcate DMRs
pcutoff value
Bumphunter DMRs
Bumphunter cutoff value
Comb-p DMRs
seed value
Probe lasso DMRs
DMR p-value cutoff
